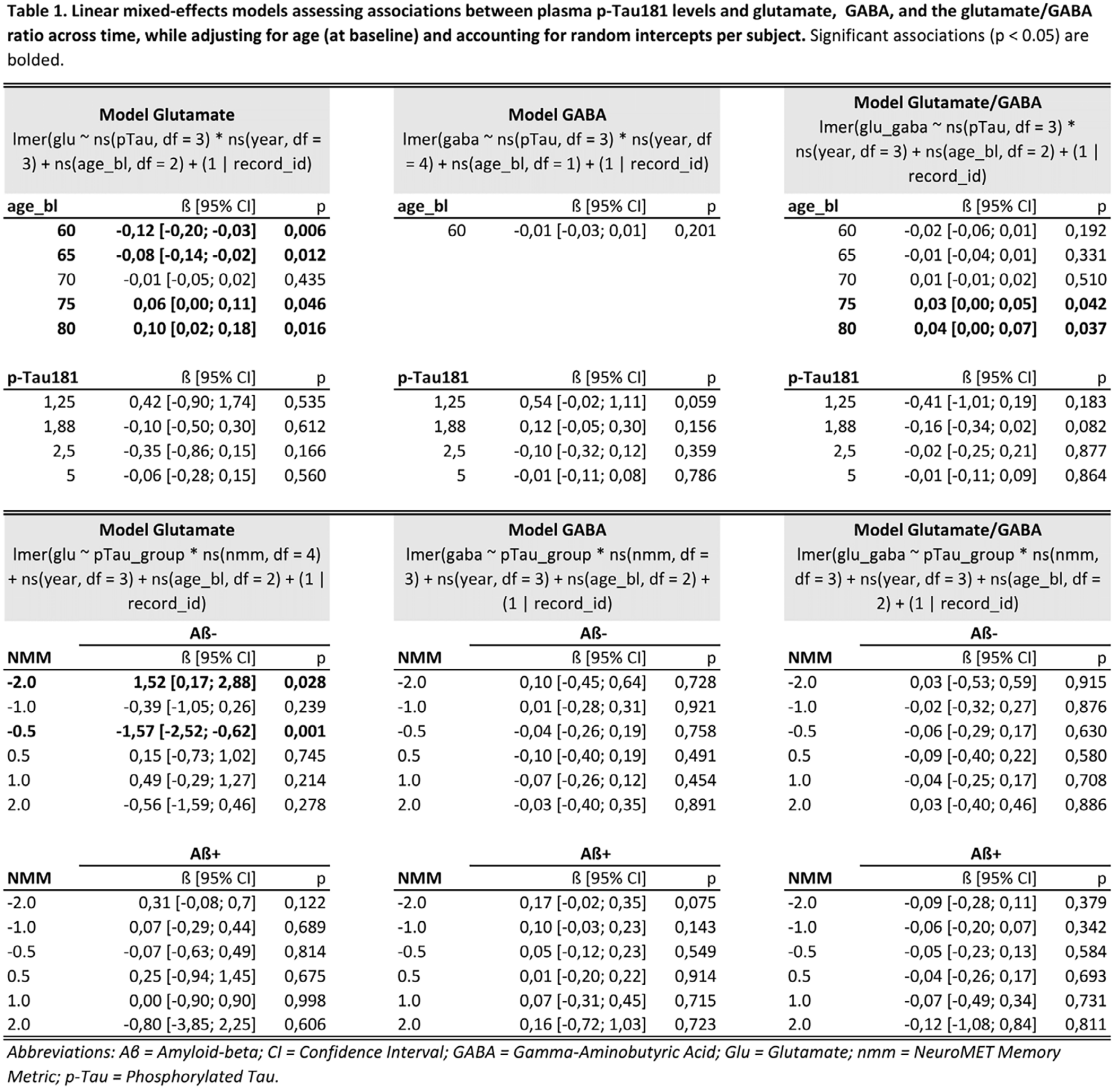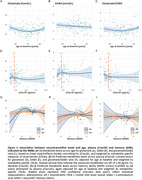# 7T MRS Glutamate and GABA in Alzheimer’s Disease

**DOI:** 10.1002/alz70861_108043

**Published:** 2025-12-23

**Authors:** Laura Göschel, Andrea Dell'Orco, Bernd Ittermann, Charlotte E. Teunissen, Patty Hoede, Jeanette Melin, Leslie Pendrill, Sebastian Roemer‐Cassiano, Nicolai Franzmeier, Peter Koertvelyessy, Agnes Flöel, Ariane Fillmer

**Affiliations:** ^1^ Charité – Universitätsmedizin Berlin, corporate member of Freie Universität Berlin and Humboldt‐Universität zu Berlin, Department of Neurology, Charitéplatz 1, Berlin Germany; ^2^ Charité – Universitätsmedizin Berlin, corporate member of Freie Universität Berlin and Humboldt‐Universität zu Berlin, NeuroScience Clinical Research Center, Charitéplatz 1, Berlin, Berlin Germany; ^3^ Charité – Universitätsmedizin Berlin, corporate member of Freie Universität Berlin and Humboldt‐Universität zu Berlin, NeuroScience Clinical Research Center, Charitéplatz 1, Berlin Germany; ^4^ Charité – Universitätsmedizin Berlin, corporate member of Freie Universität Berlin and Humboldt‐Universität zu Berlin, Department of Neuroradiology, Charitéplatz 1, Berlin Germany; ^5^ Physikalisch‐Technische Bundesanstalt (PTB), Braunschweig and Berlin Germany; ^6^ Neurochemistry Laboratory, Department of Laboratory Medicine, Amsterdam Neuroscience, Vrije Universiteit Amsterdam, Amsterdam UMC, Amsterdam Netherlands; ^7^ Neurochemistry Laboratory, Department of Laboratory Medicine, Vrije Universiteit Amsterdam, Amsterdam UMC, Amsterdam, Netherlands, Amsterdam Netherlands; ^8^ RISE, Research Institutes of Sweden, Division Safety and Transport, Measurement Science and Technology, Göteborg Sweden; ^9^ Department of Neurology, University Hospital, LMU Munich, Munich, Bavaria Germany; ^10^ Max Planck School of Cognition, Leipzig, Sachsen Germany; ^11^ Institute for Stroke and Dementia Research (ISD), University Hospital, LMU Munich, Munich, Bavaria Germany; ^12^ University of Gothenburg, The Sahlgrenska Academy, Institute of Neuroscience and Physiology, Psychiatry and Neurochemistry, Gothenburg Sweden; ^13^ Institute for Stroke and Dementia Research (ISD), LMU University Hospital, LMU Munich, Munich Germany; ^14^ Munich Cluster for Systems Neurology (SyNergy), Munich, Bavaria Germany; ^15^ Charité – Universitätsmedizin Berlin, corporate member of Freie Universität Berlin and Humboldt‐Universität zu Berlin, Department of Neurology, Charitéplatz 1, Berlin, Berlin Germany; ^16^ German Center for Neurodegenerative Diseases (DZNE), Magdeburg Germany; ^17^ University Medicine Greifswald, Greifswald, Mecklenburg‐Vorpommern Germany; ^18^ German Centre for Neurodegenerative Diseases (DZNE), Standort Rostock/Greifswald, Greifswald Germany

## Abstract

**Background:**

Functional neuroimaging studies suggest a dynamic trajectory in Alzheimer’s disease (AD), with early hyperconnectivity followed by hypoconnectivity due to pathologic progression. This study aimed to investigate this hypothesis using neurochemical markers derived from high‐field magnetic resonance spectroscopy (MRS).

**Method:**

We analyzed data from 126 older adults enrolled in the NeuroMET studies, spanning from normal aging to dementia due to suspected AD. Using ultra‐high‐field 7 Tesla MRS, we quantified levels of the neurotransmitters glutamate (excitatory) and GABA (inhibitory) in the precentral cortex. Plasma *p* ‐Tau181 was used as a proxy for AD pathology, and memory was assessed using the NeuroMET Memory Metric (NMM). A *p* ‐Tau181 threshold of >2.08 pg/mL was applied as an estimated marker of amyloid positivity (Aß+). Linear mixed‐effects models, adjusted for age at baseline, were used to evaluate associations and interaction effects.

**Result:**

Glutamate levels showed a non‐linear association with age, decreasing up to around 70 years and increasing thereafter, while GABA levels remained stable (Figure 1A–C). Before reaching the suggested pathological conversion threshold for amyloid positivity, both glutamate and GABA showed a slight, non‐significant increase in individuals with higher *p* ‐Tau181 levels (Figure 1D–F). Notably, amyloid status moderated the relationship between memory ability and glutamate levels (Figure 1G–I). Among Aß‐negative individuals, lower memory ability was associated with elevated glutamate, potentially reflecting a very early compensatory response.

**Conclusion:**

Our findings support the hypothesis of a non‐linear neurochemical trajectory in aging and early AD pathology. The observed age‐dependent fluctuations in glutamate, together with subtle shifts in response to rising plasma *p* ‐Tau181, may reflect early pathologic or compensatory mechanisms. The interaction between glutamate and memory ability, particularly in supposedly Aß‐negative individuals, suggests that excitatory neurotransmission could transiently support cognitive function in the face of emerging pathology. Notably, the threshold of plasma *p* ‐Tau181 used to define amyloid positivity may identify individuals in progressed stages, potentially missing earlier windows of therapeutic opportunity. As potential treatments may have markedly different effects depending on the stage of disease progression, investigating the trajectory of neuronal connectivity and activity is critical.